# Alien plants of Europe: introduction pathways, gateways and time trends

**DOI:** 10.7717/peerj.11270

**Published:** 2021-06-01

**Authors:** Margarita Arianoutsou, Ioannis Bazos, Anastasia Christopoulou, Yannis Kokkoris, Andreas Zikos, Sevasti Zervou, Pinelopi Delipetrou, Ana Cristina Cardoso, Ivan Deriu, Eugenio Gervasini, Konstantinos Tsiamis

**Affiliations:** 1 Faculty of Biology, National and Kapodistrian University of Athens, Department of Ecology and Systematics, Athens, Greece; 2 Nicolaus Copernicus University, Institute for the Study, Conservation and Restoration of Cultural Heritage, Toruń, Poland; 3 Faculty of Biology, National and Kapodistrian University of Athens, Department of Botany, Athens, Greece; 4 European Commission, Joint Research Centre (JRC), Ispra, Italy

**Keywords:** Alien species, Biodiversity, Plants, Europe, Pathways, Gateways, Time trends, CBD

## Abstract

The Convention on Biological Diversity (CBD) pathways classification framework used in the implementation of the European Union’s (EU) Regulation 1143/2014 on invasive alien species (IAS Regulation) has recently been adopted by the European Alien Species Information Network (EASIN), the official information system supporting the implementation of the IAS Regulation. In the current paper, the result of an alignment of the primary introduction pathways of all alien plants in Europe included in the EASIN catalogue is presented, based on the CBD framework. In total, 6,250 alien plant taxa (marine plants excluded), both alien *to* Europe (native range outside Europe) and alien *in* Europe (native range partially in Europe) are reported. Altogether 5,175 plant taxa had their primary introduction pathway aligned based on the CBD framework, while for the rest the pathway remains unknown. In addition, the taxonomy, year and country of its first record in the wild are provided for each taxon. Our analyses reveal that the main primary introduction pathways of alien plants into Europe are linked to accidental escapes from ornamental and horticultural activities. Northwestern European countries seem to act as the main gateway areas of alien plants into Europe. Recent first observations of new alien taxa growing spontaneously exhibit a contemporary accelerating trend for plants alien *to* Europe, particularly linked to ornamental and horticultural activities. On the other hand, the number of new plants alien *in* Europe seems to have stabilized over the last few decades. The present work can assist in the prioritization of introduction pathways control, with the target of slowing down the rate of alien plants introductions into Europe, following also the requirements of the IAS Regulation.

## Introduction

Alien species represent a significant risk to the receiving environments (Pejchar & Mooney, 2009; Simberloff et al., 2013; Jeschke et al., 2014; Roiloa, Yu & Barreiro, 2020). New introductions of alien species have been accelerated in recent decades by the rapid globalization, urbanization and intensification of human activities (Hulme, 2009; Roques et al., 2016; Seebens et al., 2017). Climate change and disturbance of natural ecosystems due to extreme climatic events are expected to further increase the risk of new introductions (Masters & Norgrove, 2010; Dainese et al., 2017; Dullinger et al., 2017).

According to the EU Regulation 1143/2014 on the prevention and management of the introduction and spread of invasive alien species (European Union, 2014), the term “alien species” applies to any taxa “introduced outside their natural range”, including any hybrids and varieties “that might survive and subsequently reproduce”. Invasive Alien Species (IAS) refers to species “whose introduction or spread has been found to threaten or adversely impact upon biodiversity and related ecosystem services” (European Union, 2014).

The EU Regulation 1143/2014 (hereafter referred to as the IAS Regulation) requires EU Member States to carry out a comprehensive analysis and prioritization of the pathways of introduction and spread of IAS of Union concern (European Union, 2014). In this context, databases are important for gathering, sharing and disseminating information on alien species, which are crucial for management, scientific and educational purposes. In Europe, the project Delivering Alien Species Inventories for Europe (DAISIE, 2009) represented an important milestone in creating a European database of alien species. Additionally, similar initiatives were undertaken at a regional scale, such as the NOBANIS European Network on Invasive Alien Species (NOBANIS, 2020) and the East and South European Network for Invasive Alien Species (ESENIAS, 2020). It is noteworthy to report also Global datasets, such as the Global Biodiversity Information Facility (GBIF, 2020) and the CABI-Invasive Species Compendium (CABI, 2020), which include records of alien species in Europe.

Aiming to aggregate and integrate existing information on alien species in Europe, the European Alien Species Information Network (EASIN, 2020) has been developed by the European Commission’s Joint Research Centre (Katsanevakis et al., 2012). EASIN acts as a single aggregation point for sharing and disseminating information, where available knowledge on alien species from various data sources is standardized, harmonized and integrated (Katsanevakis et al., 2015; Deriu et al., 2017). EASIN aims to underpin alien species related policy and management decisions (Katsanevakis et al., 2015), and constitutes the core of the information system supporting EU Member States in the implementation of the IAS Regulation (European Union, 2014; Art. 25).

Identification of key-recipient regions of alien introductions is essential, since it may help to predict, prevent, and control future introductions, pinpointing geographical areas where management should be focused (Vermeij, 1996). In addition, since most alien species are extremely difficult to control after their establishment (Genovesi, 2005; Vilà et al., 2010), scientific effort should be directed at finding appropriate means to prevent their entry into new areas, by managing their potential pathways (Hulme, 2009; Hulme, 2015; McGeoch et al., 2016; Ojaveer et al., 2018; Tsiamis et al., 2020).

Pathway management is an essential aspect in tackling biological invasions and can benefit from applying a consistent classification, hierarchy, and terminology (Essl et al., 2015). To aid this effort, a standardized pathways terminology and hierarchical classification was proposed by Hulme et al. (2008) and largely adopted by EASIN in 2012 (Katsanevakis et al., 2012). The framework set by Hulme et al. (2008) has been extensively used in various studies assessing introduction pathways across different environments and taxonomic groups (Katsanevakis et al., 2013; Roques et al., 2016; Pergl et al., 2017).

The global increasing threat of alien species has led the Convention on Biological Diversity (CBD) to a comprehensive review of introduction pathways (CBD 2014), which aims to improve the understanding and the consistency of those pathways. This review has become a global standard of terminology for introduction pathways and a key requirement for interoperability and harmonization of databases (Deriu et al., 2017; Groom et al., 2017; Groom et al., 2019; Saul et al., 2017), risk analysis and large-scale studies (Pergl et al., 2017; Tsiamis et al., 2018). The CBD pathways classification framework includes six principal pathway categories (Release, Escape, Transport-contaminants, Transport-stowaway, Corridors, and Unaided), most of them hosting several subcategories (44 in total).

The CBD classification framework is used in the implementation of the IAS Regulation and was recently adopted by EASIN. To this end, EASIN aligned its pathways’ information with the CBD framework, including taxa from various taxonomic groups and habitats (Tsiamis, Cardoso & Gervasini, 2017; Pergl et al., 2020). However, the bulk of the information on the alien plant pathways included in EASIN (> 5,500 taxa) until recently was not aligned with the CBD categorization. This paper aims to fill this gap, presenting basic information on all alien plant taxa included in EASIN and their pathways after their alignment with the CBD scheme. More specifically, the objectives of the current work are: (a) to highlight the main introduction pathways of alien plants in Europe, (b) to explore the first records in the wild in Europe (i.e., not in cultivation or nurseries, hereinafter called as “the wild”) by assessing which countries act as the major gateways, and (c) to investigate the temporal trends of first records of new alien plants and related pathways, with emphasis on the more recent decades. Such analysis can assist the prioritization of pathways management, which can contribute to slowing the rate of alien species introductions into Europe’s wild, in line with the targets of the IAS Regulation.

## Materials & Methods

### Pan-European inventory

For the present analysis, we used the catalogue of alien plant taxa (species, subspecies and hybrids) reported in Europe, as updated by EASIN until the end of 2019 (Table S1). Only vascular plants (Tracheophyta) have been included in the analysis. Mosses (Bryophyta), liverworts (Marchantiophyta), marine vascular plants and algae were not taken into account. Alien plant taxa correspond to records found in the wild as spontaneous (casual or established/naturalized). Alien plants that have been found only in cultivation and have never been observed growing wild were excluded.

The EASIN catalogue of alien plants is mainly based on information retrieved from DAISIE (45% contribution to the EASIN records), NOBANIS (29%), (Online Atlas of the British and Irish flora, 2018) (8%) and Manual of the Alien Plants of Belgium (Verloove, 2020) (6%). The remaining EASIN plant records originate from several other global, European, regional and national databases (Table S2) and scientific publications.

The EASIN catalogue contains taxa which are considered as (a) alien *to* Europe (i.e., taxa with a native range outside Europe, as defined by Lambdon et al., 2008), (b) alien *in* Europe (i.e., taxa with native range partially in Europe, as defined by Lambdon et al., 2008), (c) cryptogenic (i.e., taxa with no definite evidence of their alien or native status in Europe), and (d) questionable (i.e., taxa whose presence in Europe is uncertain, possible misidentifications, as well as taxa with unresolved taxonomic status). A taxon is included in the inventory if it is considered as alien in at least one European country, including the European part of the Macaronesia (the Azores, Canary Islands, Madeira). In order to standardize the nomenclature, World Flora Online (World Flora Online (WFO), 2020) was mainly used. The Euro+Med (2020), the Plants of the World Online (Plants of the World Online (POWO), 2020; The Plant List, 2020) and the National Biodiversity Network of the UK (National Biodiversity Network (NBN), 2020) were additionally used. For the subsequent analyses, only alien taxa *to* and *in* Europe were considered, while cryptogenic and questionable taxa were excluded.

*Primary pathways*: The classification of pathways for alien plants’ primary introduction followed the CBD classification framework (CBD, 2014; Hulme et al., 2008), and the technical guide of application by Harrower et al. (2017). This guide provided additional clarifications on the distinction between CBD pathways, for example between the subcategories “Horticulture” and “Ornamental purpose other than horticulture”. The former applies to plants that have escaped from commercial facilities (nurseries, greenhouses) or during the transport of horticultural products. The latter applies when the escape occurs from landscaped habitats or plant collections, excluding botanical gardens, which is a distinct subcategory.

Original information on pathways was based on data from DAISIE, NOBANIS, EPPO and other sources as provided in Table S3. For 8% of the taxa, expert judgment was used for pathway assignment based on CBD categorization. For several taxa, the pathway assignment could be decided only for the main CBD pathway categories but not for the subcategories, due to lack of adequate information. To this end, we have added the following subcategories:

1. Release in nature: unknown;
2. Escape from confinement: unknown;
3. Transport-contaminant: unknown;
4. Transport-stowaway: unknown.

*Gateways*: based on the information of the main sources appearing in Table S2, the countries of the first observations of alien plants in the wild were identified (hereafter referred to as gateway countries). Taxa first found from Macaronesia were assigned to the corresponding country (e.g., if a taxon was first found in the Canary Islands the gateway country would be Spain).

*Time-trends*: the year of the first record of an alien plant in the wild was used as the best available estimate of the year of its initial occurrence. In the case of taxa alien *in* Europe, the first record in a European country outside its native range was used.

*Data analyses*: pathway patterns were analyzed separately for taxa alien *to* Europe, and taxa alien *in* Europe. In addition, archaeophytes (i.e., alien taxa that arrived in Europe before 1500 AD) and neophytes (i.e., taxa that arrived after 1500 AD) were also distinguished based on Pyšek et al. (2004). For several taxa there was high uncertainty on the gateways and time-trends of the first record in the wild due to lack of data, especially concerning old records. Therefore, detailed analyses for the main gateway countries per taxonomic group (Families) were performed for alien plants of Europe recorded for the first time in the wild after 1950, for which the uncertainty level was lower. To simplify the graphic analyses, we grouped introduction pathways with a minor contribution to the generic group “Rest pathways”. Several taxa were linked to more than one CBD introduction pathway subcategory. To avoid double-counting of species for a specific gateway country or Family a value of 1/k was provided for each of the k associated pathways so that the overall contribution of each species to each gateway country or Family is always equal to 1.

## Results

### Pan-European inventory

In total, 6,250 alien, 8 cryptogenic and 24 questionable plant taxa have been reported as spontaneous in the wild by 2019. Among the alien taxa, 3,021 are alien *to* Europe and 3,229 are alien *in* Europe, both cases including naturalized and casual taxa. The Families with the higher numbers of alien taxa are Asteraceae (11.4%), Poaceae (9.7%), Fabaceae (6.8%), Rosaceae (6.4%), and Brassicaceae (4%).

### Primary pathways of introduction

Primary pathways of introduction of alien plants into Europe were assigned to 5,175 taxa, while for the remaining taxa the pathway was marked as “Unknown” since no relevant published information was found.

**Figure 1:**
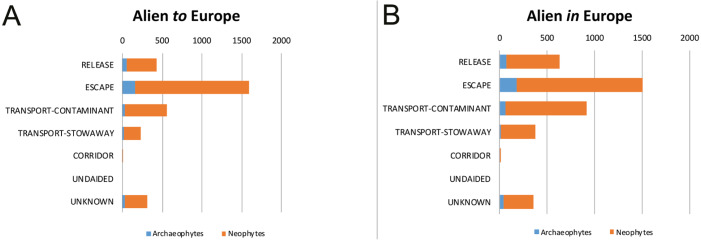
CBD principal introduction pathways for alien plants of Europe. The number of alien plants recorded in the wild, shown separately for taxa alien *to* Europe, i.e., taxa with a native range outside Europe (A), and for taxa alien *in* Europe, i.e., taxa with native range partially in Europe (B). Distinction between archaeophytes and neophytes is also provided. Several taxa are linked to more than one pathway.

Regarding both taxa alien *to* Europe and *in* Europe, dominant pathways correspond to the main CBD categories “Escape”, “Transport-Contaminant” and “Release” (Fig. 1).

**Figure 2:**
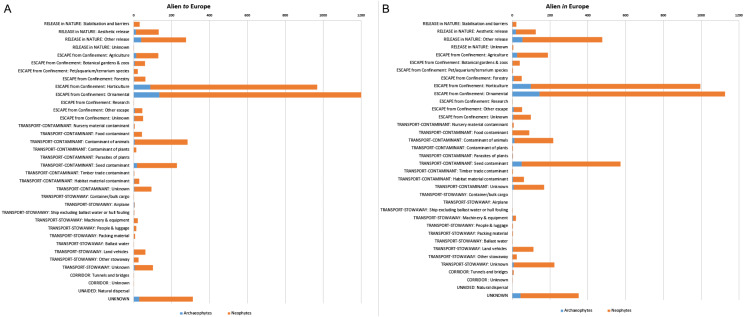
CBD subcategory introduction pathways for alien plants of Europe. The number of alien plants recorded in the wild, shown separately for taxa alien *to* Europe, i.e., taxa with a native range outside Europe (A), and for taxa alien *in* Europe, i.e., taxa with native range partially in Europe (B). Distinction between archaeophytes and neophytes is also provided. Several taxa are linked to more than one pathway.

When it comes to the CBD subcategories, the highest number of taxa alien *to* Europe were associated with “Escape from confinement: ornamental” (hereafter referred to as ornamental), “Escape from confinement: horticulture” (hereafter referred to as horticulture), “Transport-contaminant: contaminant on animals”, “Release in nature: other release”, and “Transport-contaminant: seed contaminant” (hereafter referred to as seed contamination) (Fig. 2A). A similar pattern is shown by the taxa alien *in* Europe although the pathway “Transport-contaminant: contaminant on animals” has a minor role in alien taxa introductions compared to the aforementioned pathways (Fig. 2B). We should note that several CBD subcategory pathways were associated with very low numbers of plant introductions, both for taxa alien *to* Europe and taxa alien *in* Europe.

### Gateway countries

The gateway countries were identified for 5,746 taxa. Overall, for taxa alien *to* Europe, the most important gateway countries are the United Kingdom, Belgium, Portugal and Austria (Fig. S1). Considering taxa alien *in* Europe, the top-ranking countries are the United Kingdom, Sweden, Belgium and Denmark (Fig. S1).

**Figure 3:**
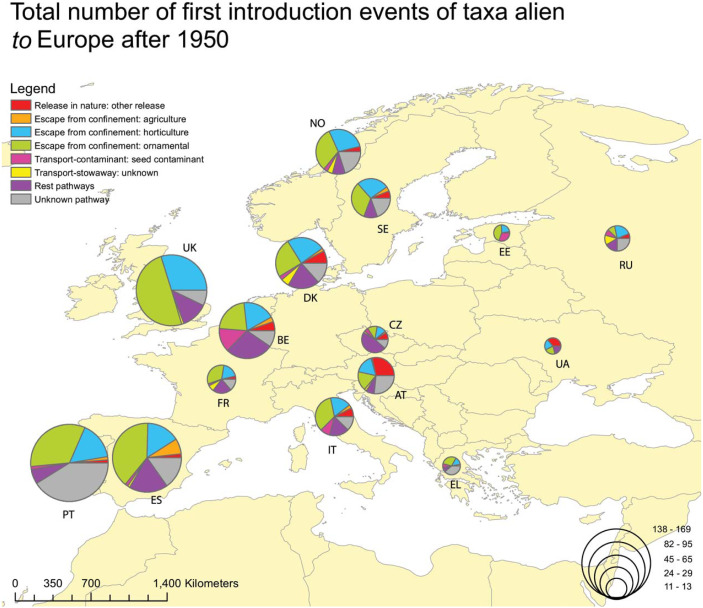
Total number of plants alien *to* Europe firstly recorded in the wild after 1950, per gateway country, and associated with the CBD introduction pathway. Proportion of plants alien *to* Europe firstly recorded in the wild after 1950, through different CBD subcategory introduction pathways, per gateway country (i.e., countries of first observation in the wild). For clarity, data is shown for countries with more than 10 first records of alien plants. Taxa linked to more than one pathway were given a value of 1/k for each of the k associated pathways so that the overall contribution of each taxon to the total number of new arrivals was always 1.

**Figure 4:**
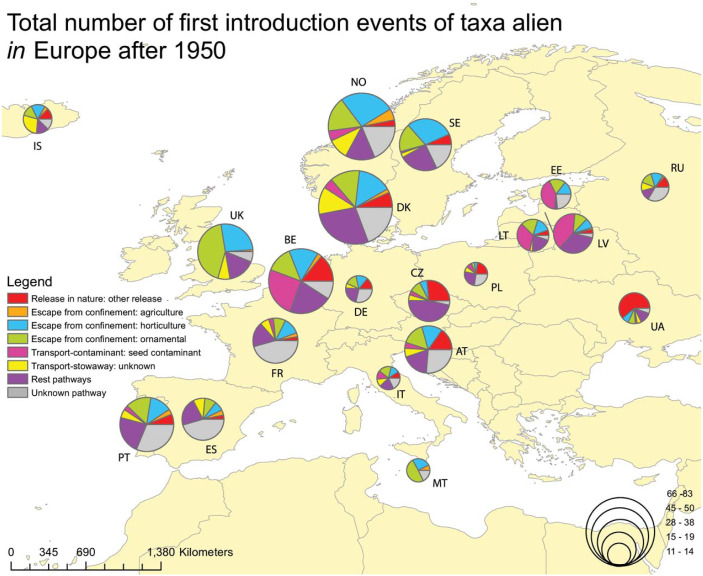
Total number of plants alien *in* Europe firstly recorded in the wild after 1950, per gateway country and associated with the CBD introduction pathway. Proportion of plants alien *in* Europe firstly recorded in the wild after 1950, through different CBD subcategory introduction pathways, per gateway country (i.e., countries of first observation in the wild). For clarity, data is shown for countries with more than 10 first records of alien plants. Taxa linked to more than one pathway were given a value of 1/k for each of the k associated pathways so that the overall contribution of each taxon to the total number of new arrivals was always 1.

When focusing on alien plants recorded for the first time in the wild after 1950, the higher numbers of alien taxa *to* Europe were reported from Portugal, the United Kingdom, Spain and Belgium, and they were related to a large extent with ornamental and horticulture escapes (Fig. 3). For taxa alien *in* Europe firstly recorded after 1950, the higher numbers were reported from Denmark, Norway, Belgium and the United Kingdom. Again, the most common pathways were escapes from ornamental and horticulture (Fig. 4). The seed contamination pathway was relatively high for Belgium.

### Time-trends

**Figure 5:**
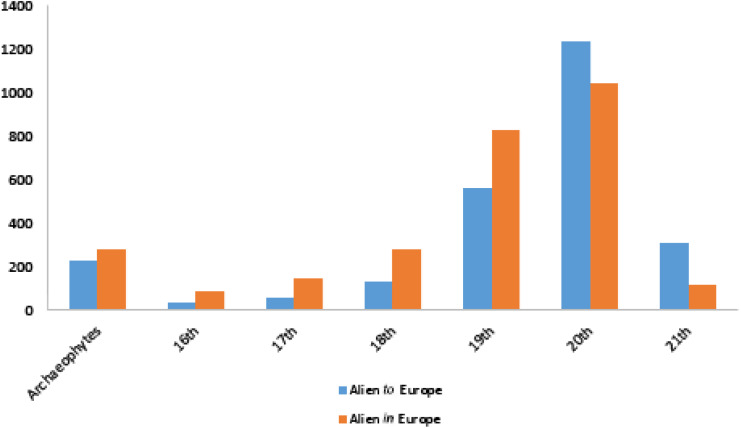
Time trends of alien plants firstly recorded in the wild of Europe. Number of first records of alien plants reported in the wild, provided per century, and for both taxa alien *to* Europe and taxa alien *in* Europe.

The year of the first record of an alien plant in the wild (or in areas outside their native distribution for taxa alien *in* Europe) was documented for 5,296 taxa. Among the alien taxa, 506 taxa are archaeophytes while the remaining are neophytes. The rate of newly recorded alien plants *to* European wild is constantly increasing from the 16th to the 20th century (Fig. 5). When it comes to taxa alien *in* Europe, this rate was also accelerating from the 16th up to the 19th century, while slowing down in the 20th century (Fig. 5).

**Figure 6:**
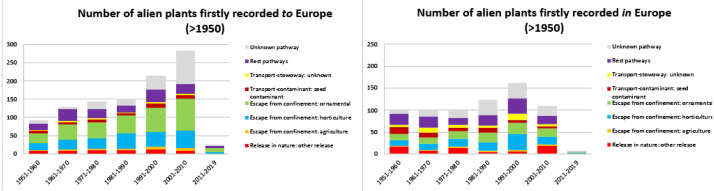
Number of alien plants firstly recorded in the wild of Europe after 1950, and associated pathways per decade. Number of alien plants firstly recorded in the wild of Europe after 1950, given per decade and associated to the relevant CBD subcategory introduction pathways, provided for taxa alien *to* Europe (A) and taxa alien *in* Europe (B). Taxa linked to more than one pathway were given a value of 1/k for each of the k associated pathways so that the overall contribution of each taxon to the total number of new arrivals was always 1.

Focusing on alien plants observed for the first time after 1950, the number of taxa alien *to* Europe shows an increasing trend during the recent decades, except for the period 2011–2019 (Fig. 6A). On the other hand, the number of first records of new alien plants *in* Europe seems to be stable during the same period (Fig. 6B), with the exception again of the very low number reported for the period 2011–2019. For taxa alien *to* Europe, the main CBD introduction pathways subcategories are associated with escapes from ornamental and horticulture (Fig. 6A). For taxa alien *in* Europe, introduction pathways appear more diverse during the last decades (Fig. 6B).

**Figure 7:**
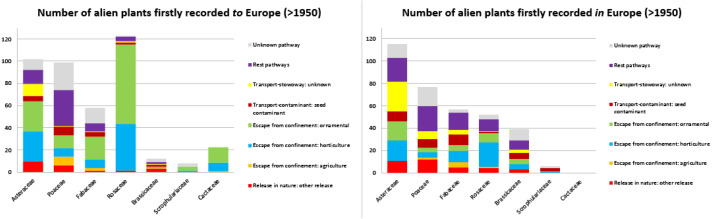
Number of alien plants firstly recorded in the wild of Europe after 1950, per main Family and associated introduction pathways. Number of alien plants firstly recorded in the wild after 1950, given per main Family and associated to the relevant CBD subcategory introduction pathways, provided for taxa alien *to* Europe (A) and taxa alien *in* Europe (B). Taxa that were linked to more than one pathway were given a value of 1/k for each of the k associated pathways so that the overall contribution of each taxon to the total number of new arrivals was always 1.

The taxonomic analysis at the Family level reveals that Rosaceae, Asteraceae, Poaceae and Fabaceae, contribute with the highest numbers of first records of alien plants after 1950 *to* Europe. Ornamental purposes and horticulture are their main introduction pathways, except for Poaceae** for which the main pathways either vary or are unknown (Fig. 7A). For plants that appeared after 1950 *in* Europe, the Families hosting the highest numbers are Asteraceae, Poaceae, Fabaceae, Rosaceae** and Brassicaceae. Patterns of pathways are not consistent across Families (Fig. 7B).

## Discussion

Alien plants constitute a major group of alien species reported for Europe (EASIN, 2020). In addition, Europe (together with America) is the continent with the highest numbers of alien plant species across the globe (Van Kleunen et al., 2015; Pyšek et al., 2017). It should also be mentioned that by 2020, out of the 66 invasive alien species included in the list of IAS of Union concern, 36 (55%) are plants.

There are several studies on alien plants in Europe, focusing on one or few species (e.g., Moravcova et al., 2006; Perglova, Pergl & Pyšek, 2006; Kollmann, Banuelos & Nielsen, 2007) or checklists at a local, regional or country-level (e.g., Boršić et al., 2008; Arianoutsou et al., 2010; Stešević & Petrović, 2010; Medvecka et al., 2012; Petrova, Vladimirov & Georgiev, 2013; Barina et al., 2014; Maslo, 2016; Gudžinskas, Petrulaitis & Žalneravičius, 2017; Bomanowska et al., 2019; Burda & Koniakin, 2019). Extended inventories covering alien plant taxa have been undertaken by DAISIE at a European scale (Lambdon et al., 2008; Pyšek et al., 2009), and by the Global Naturalized Alien Flora (GLONAF) and the Global Register of Introduced and Invasive Species (GRIIS) at a global scale (van Kleunen et al., 2015; Pyšek et al., 2017; Pagad et al., 2018).

Pyšek et al. (2017) reported 4,139 alien plant taxa as naturalized in Europe. In the current study, a considerably higher number of taxa (2,111 more) is reported, which can be attributed to the inclusion of both naturalized and casual taxa, as well as taxa reported from Macaronesia and the European Caucasian area, that is Georgia, Armenia etc.

The analysis of introduction pathways following the CBD categorization revealed that the dominant pathway is “Escape”, which is also reported for biological invasions at a global scale (Essl et al., 2015). At the subcategory level, the most common pathways are escapes associated with ornamental and horticultural activities, both for taxa alien *to* and *in* Europe. The same pattern was previously reported by several studies in Europe (Bell, Wilen & Stanton, 2003; Dehnen-Schmutz et al., 2007a; Dehnen-Schmutz et al., 2007b; Lambdon et al., 2008; van Kleunen et al., 2018; Aymerich & Sáez, 2019). Intentional release for various purposes and transport through contaminants on animals are also important pathways for alien plants into Europe. Moreover, seed contamination should be highlighted as one of the main pathways, in particular for taxa that are alien *in* Europe, in line with the results of similar studies in Europe (Ferus et al., 2015; Cossu et al., 2019) and outside the continent (Wilson et al., 2016; Oseland et al., 2020).

NW European countries are serving as gateway countries for a high number of alien plants. This should be attributed to a long history in horticulture, gardening, ornamental plant trade and relationships with European overseas territories. This is particularly true for countries with a long tradition in botanical research, and landscape architecture, such as the United Kingdom, Belgium, Sweden and Austria. Better sampling effort could also be encountered as one of the reasons (van Kleunen et al., 2015; Celesti-Grapow et al., 2016). During the last decades (>1950), other countries also act as remarkable gateways, such as Spain and Portugal for taxa alien *to* Europe. However, this should be related to recent findings in the Macaronesia area due to an intensified monitoring effort (e.g., Silva, Ojeda Land & Rodrıguez Luengo, 2008; Verloove et al., 2017a; Verloove et al., 2017b) and to several “acclimatisation gardens” (e.g., the Acclimatisation Gardens of La Orotava in the Canary Islands). Similarly, Denmark and Norway are reported as important gateways for alien taxa *in* Europe after 1950, which could also be the result of increased sampling effort and availability of information (e.g., via the NOBANIS database). On the other hand, the southern European countries (except for Italy) do not seem to act as important gateways, but this could be related to the scarcity of relevant studies. The latter stresses the need for enhanced sampling and availability of related data across Europe.

Time trends reveal an accelerating rate of first observations of new alien taxa in the wild since the 16th century (see also Lambdon et al., 2008), a result that should be attributed to the increased urbanization and international trade coupled with an intensification in the recording efforts. The number of plants firstly recorded in the wild as alien *to* Europe exhibits a remarkable increase in the 20th century, and a constant increase between 1951 and 2010, coupled with an increasing trend of plant escapes associated with ornamental and horticulture pathways. These findings are in agreement with the results of previous studies (e.g., Pyšek et al., 2003; Walker, 2007; Pyšek et al., 2009; Seebens et al., 2017; Aymerich & Sáez, 2019). On the other hand, the rate of first observations of alien taxa *in* Europe decreases in the 20th century and becomes rather stable during the period 1951-2010.

For both taxa alien *to* and *in* Europe, the number of first observations in the wild significantly drops during the last decade (2011–2019). However, we believe that this fall in numbers should be attributed to the time lag between the occurrence of a taxon in the wild and the time of its reporting (see also Crooks, 2005; Rouget et al., 2016; Smith et al., 2018; Zenetos et al., 2019). It is possible that information on observations of new alien taxa could be still unpublished or in local repositories, thus remaining absent in several databases and scientific literature.

Since the 1950s, Asteraceae, Poaceae, Fabaceae and Rosaceae are amongst the richest Families in numbers of first observations of alien plants, as reported also by Arianoutsou et al. (2013) for the Mediterranean biome and Pyšek et al. (2017) at a global level. Similar patterns are also observed in checklists of alien flora at country and European level (e.g., Lambdon et al., 2008; Celesti-Grapow et al., 2009; Celesti-Grapow et al., 2016; Arianoutsou et al., 2010; Pyšek et al., 2012; Petrova, Vladimirov & Georgiev, 2013; Del Guacchio & La Valva, 2018; Uludag et al., 2017; Galasso et al., 2018). Asteraceae, Poaceae, and Fabaceae are mostly represented by herbaceous plants expected to be more easily dispersed and become established in new environments, while Rosaceae includes trees and woody shrubs commonly used as ornamental plants and in horticulture (see also Pyšek et al., 2017). Cactaceae are also important for plants of ex-European origin reported in islands, such as the Macaronesia (Otto & Verloove, 2016; Verloove, Salas-Pascual & MarreroRodrıguez, 2018; Verloove, 2019), and the Mediterranean Islands (Celesti-Grapow et al., 2016).

Following the recently observed trend, the number of alien plant taxa of Europe is likely to further increase in the near future, especially due to new arrivals of taxa non-native *to* Europe and associated with ornamental and horticulture purposes. This reveals and stresses a priority need for more effective control of these kinds of plants and their pathways. Increased attention should be given to Families with the higher numbers of new taxa appearing after 1950 (e.g., Rosaceae, Asteraceae, Cactaceae). According to Article 11 of the IAS Regulation, Member States must carry out a comprehensive analysis of the introduction pathways of IAS in their territory and identify those that require priority action. The current paper can contribute to this action by providing the top-priority primary pathways of alien plants into Europe from 1950 to date, which can be crucial for effective prevention and appropriate management (Brunel et al., 2010; McGeoch et al., 2016). In addition, the information provided on the CBD introduction pathways can support the risk assessments required by the IAS regulation (Roy et al., 2018), for prioritization and pest risk analysis following IPPC/EPPO standards (Brunel et al., 2010), as well as for horizon-scanning exercises (Roy et al., 2019; Tsiamis et al., 2020).

When it comes to the CBD categorization framework, the vast majority of alien plants have been associated with a few specific pathways compared to the total of the CBD subcategory pathways. Several CBD pathways were not assigned at all (e.g., Release in nature: fishery in the wild) or were relevant for only a few taxa (e.g., Corridor: tunnels and bridges). It should be noted, however, that the accurate determination of an alien species’ pathway is not always an easy task due to the limited evidence available, and it may be characterized by high levels of uncertainty (Katsanevakis et al., 2013; Pergl et al., 2020), setting the need for experts’ judgment (Essl et al., 2015). Indeed, for several taxa it was impossible to conclude on a specific CBD subcategory or the introduction pathway was completely unknown.

Applying the CBD pathways subcategories to alien plants was not always straightforward because of the overlap between pathway subcategories. For example, during the current alignment exercise, it was not always clear which plant introductions corresponded to horticulture or ornamental activities or both. Some CBD pathways are too broad, such as “Transport-contaminant: contaminant on animals”, which can refer to activities related to the breeding of animals and trade, but mainly with plant seeds attached to their fur. In addition, in the scientific literature, the introduction pathways were described with various terms (e.g., gardening; van Kleunen et al., 2018), which can correspond to multiple CBD subcategories. Therefore, several CBD subcategories would need further clarification (e.g., the distinction between the horticulture and ornamental pathways) and we would stress the need for commonly accepted definitions and related interpretations of the CBD pathways, ensuring consistent alignment outcomes (see also Tsiamis, Cardoso & Gervasini, 2017). Towards that aim, Pergl et al. (2020) have recently proposed specific amendments that could improve the interpretation and clarity of the pathway categories which can lead to a better use of the CBD pathways categorization. For example, Pergl et al. (2020) have further clarified the distinction between ornamental and horticulture pathways, which is based on the escapes from gardens and other landscaped habitats (ornamental) versus the escapes from commercial, industrial facilities (horticulture).

## Conclusions

Our research shows that the dominant pathways of primary introductions of alien plants into Europe are linked with accidental escapes from ornamental and horticultural activities. Northwestern European countries seem to act as the main gateway countries for alien plants, although biases such as the monitoring effort should be taken into account. Recent first records of alien plants in the wild exhibit a contemporary accelerating trend for plants alien *to* Europe, particularly linked with ornamental and horticulture pathways. On the other hand, the number of alien plants *in* Europe seems to be stabilized during the last decades.

The present work can assist in the prioritization of pathways management, aiming to slow down the rate of new alien plant introductions into Europe, following also the requirements of the IAS Regulation.

The availability of related data on alien plants of Europe is dynamic; knowledge gaps need to be addressed and more information will come to light by future studies, in particular on species’ introduction pathways. Pathways used in plant introductions are also dynamic through time. Increased arrivals could be expected in the near future, due to the need for species used in forestry, agriculture and as biofuel that are more adaptable to climate change.

Finally, it should be highlighted that the current work focused exclusively on the primary introduction pathways of alien plants into Europe. However, addressing the secondary pathways of their dispersal within Europe is also crucial to avoid further spread to other countries.

##  Supplemental Information

10.7717/peerj.11270/supp-1 Supplemental Information 1 Alien plant taxa in the wild of Europe based on the EASIN Catalogue List of alien plant taxa (species, subspecies and hybrids) of Europe as included in the EASIN Catalogue by 2019. Information on taxa authorities, category, year and country of first record into the wild of Europe, environment, taxonomic tree, taxa alien *in* Europe and pathways based on CBD are provided for each taxon. Mosses (Bryophyta), liverworts (Marchantiophyta), marine vascular plants and algae were not considered.

10.7717/peerj.11270/supp-2 Supplemental Information 2 Sources of the EASIN dataset of alien plants of Europe Global, European, regional and national databases from where information was extracted for creating the EASIN dataset on alien plants of Europe (see Table S1 ), including information on year and gateway country of first record into Europe’s wild of each alien taxon.

10.7717/peerj.11270/supp-3 Supplemental Information 3 Sources of pathway information of alien plants of Europe Global, European, regional and national databases as well as scientific articles from where information was extracted for assigning primary pathways for the first records of alien plants into Europe’s wild (see Table S1).

10.7717/peerj.11270/supp-4 Supplemental Information 4 Number of alien plants firstly recorded in the wild of Europe per main gateway countries Number of alien plants firstly recorded in the wild of Europe per main gateway country, provided for taxa alien *to* Europe and taxa alien *in* Europe. For clarity, data is shown for gateway countries with more than 50 new arrivals.

## Additional Information and Declarations

### Data Availability

The following information was supplied regarding data availability: The raw data are available as a Supplemental File.
