# Supplementary material for: Alien plants of Europe: introduction pathways, gateways and time trends
Source: PeerJ. 2021 Jun 1;9:e11270. doi: 10.7717/peerj.11270 (PMC8176916; doi:10.7717/peerj.11270)
Supplement: Supplemental Information 2 — Global, European, regional and national databases from where information was extracted for creating the EASIN dataset on alien plants of Europe (see Table S1 ), including information on year and gateway country of first record into Europe’s wild of each alien taxon. [file peerj-09-11270-s002.docx]

**Supplementary Table 2.** Global, European, regional and national databases from where information was extracted for creating the EASIN dataset on alien plants of Europe (see *Table S1*), including information on year and gateway country of first record of each alien taxon into Europe's wild.

| Sources | URL | % contribution in EASIN dataset |
| --- | --- | --- |
| DAISIE-Delivering Alien Invasive Species Inventories for Europe | http://www.europe-aliens.org/default.do | 45 |
| NOBANIS - European Network on Invasive Alien Species | www.nobanis.org | 29 |
| Online Atlas of the British and Irish flora | http://www.brc.ac.uk/plantatlas/ | 8.5 |
| Manual of the Alien Plants of Belgium | <http://alienplantsbelgium.be/> | 6 |
| Euro+Med PlantBase - the information resource for Euro-Mediterranean plant diversity. | http://ww2.bgbm.org/EuroPlusMed/ | 4 |
| EPPO-European and Mediterranean Plant Protection Organization | https://www.eppo.int/INVASIVE_PLANTS/ias_plants.htm | 2 |
| POWO-Plants of the World Online | http://www.plantsoftheworldonline.org/ | 1 |
| CABI-Invasive Species Compendium. Datasheets, maps, images, abstracts and full text on invasive species of the world | http://www.cabi.org/isc | 1 |
| Goberno de Canarias. Especies introducidas en Canarias | http://www.interreg-bionatura.com/especies/index.php | 0.5 |
| GBIF \| Global Biodiversity Information Facility | https://www.gbif.org/ | 0.5 |
| BioDat Database: Data on invasive plants in Russia | http://biodat.ru/db/intro/plant_e.htm | 0.2 |
| Flora of Iceland: Updated checklist of the flowering plants and fens of Iceland | http://www.floraislands.is/PDF-skjol/plontutal.pdf | 0.1 |
| Ecological Flora of the British Islands | http://www.ecoflora.co.uk/search_aliens05.php?plant_no=1950080020 | <0.1 |
| Global Invasive Species Database | www.issg.org | <0.1 |
| Invasive species in Belgium | http://ias.biodiversity.be/ | <0.1 |
| National Biodiversity Network - NBN Gateway | https://data.nbn.org.uk/Taxa | <0.1 |
| NNSS - GB non-native species secretariat | http://www.brc.ac.uk/gbnn_admin/ | <0.1 |
| Published scientific literature |  | 2 |
