# Supplementary material for: Alien plants of Europe: introduction pathways, gateways and time trends
Source: PeerJ. 2021 Jun 1;9:e11270. doi: 10.7717/peerj.11270 (PMC8176916; doi:10.7717/peerj.11270)
Supplement: Supplemental Information 3 — Global, European, regional and national databases as well as scientific articles from where information was extracted for assigning primary pathways for the first records of alien plants into Europe’s wild (see Table S1). [file peerj-09-11270-s003.docx]

**Supplementary Table 3.** Global, European, regional and national databases as well as scientific articles from where information was extracted for assigning primary introduction pathways for alien plants of Europe (see *Table S1*).

| Sources | URL | % contribution in EASIN dataset |
| --- | --- | --- |
| DAISIE-Delivering Alien Invasive Species Inventories for Europe | http://www.europe-aliens.org/default.do | 25 |
| Saul W-C, Roy HE, Carnevali L, Genovesi P, Harrower CA, Hulme PE, Pagad S, Pergl J, Jeschke JM. 2017. Assessing patterns in introduction pathways of alien species by linking major invasions data. *Journal of Applied Ecology* 54: 657-669. |  | 16 |
| NOBANIS - European Network on Invasive Alien Species | www.nobanis.org | 11 |
| Manual of the Alien Plants of Belgium | alienplantsbelgium.be/ | 10 |
| Verloove F. 2006. Catalogue of neophytes in Belgium (1800-2005). *Scripta Botanica Belgica* 39: 89. |  | 6 |
| GBIF \| Global Biodiversity Information Facility | https://www.gbif.org/ | 6 |
| EPPO-European and Mediterranean Plant Protection Organization | https://www.eppo.int/INVASIVE_PLANTS/ias_plants.htm | 4 |
| Online Atlas of the British and Irish flora | http://www.brc.ac.uk/plantatlas/ | 4 |
| Euro+Med PlantBase - the information resource for Euro-Mediterranean plant diversity. | http://ww2.bgbm.org/EuroPlusMed/ | 1.5 |
| Royal Horticultural Society website | https://www.rhs.org.uk/Plants | 1 |
| ILDIS - The International Legume Database & Information Service | http://www.ildis.org/ | 1 |
| POWO-Plants of the World Online | http://www.plantsoftheworldonline.org/ | 0.5 |
| Pladias. Database of the Czech flora and vegetation | https://pladias.cz/en/taxon | 0.3 |
| CABI-Invasive Species Compendium. Datasheets, maps, images, abstracts and full text on invasive species of the world | http://www.cabi.org/isc | 0.3 |
| Plants Database. The National gardening association | https://garden.org/plants/ | 0.2 |
| National Biodiversity Network - NBN Gateway | https://data.nbn.org.uk/Taxa | 0.2 |
| Goberno de Canarias. Especies introducidas en Canarias | http://www.interreg-bionatura.com/especies/index.php | <0.1 |
| Flora of Iceland: Updated checklist of the flowering plants and fens of Iceland | http://www.floraislands.is/PDF-skjol/plontutal.pdf | <0.1 |
| Ecological Flora of the British Islands | http://www.ecoflora.co.uk/search_aliens05.php?plant_no=1950080020 | <0.1 |
| Other scientific literature |  | 5 |
| Expert opinion |  | 8 |
